# n-3 PUFA Induce Microvascular Protective Changes During Ischemia/Reperfusion

**DOI:** 10.1007/s11745-014-3961-0

**Published:** 2014-10-26

**Authors:** Maria das Graças Coelho de Souza, Cristiane Maria Simonato Conde, Camila Maurente Laflôr, Fernando Lencastre Sicuro, Eliete Bouskela

**Affiliations:** Laboratório de Pesquisas Clínicas e Experimentais em Biologia Vascular, Centro Biomédico, Universidade do Estado do Rio de Janeiro, Rua São Francisco Xavier, 524, Pavilhão Reitor Haroldo Lisboa da Cunha térreo, Rio de Janeiro, RJ 20550-013 Brazil

**Keywords:** n-3 PUFA, Microvascular diameter, Leukocyte rolling/sticking, Macromolecular permeability, Ischemia/reperfusion, Hamster cheek pouch

## Abstract

Ischemia/reperfusion (I/R) injury can occur in consequence of myocardial infarction, stroke and multiple organ failure, the most prevalent cause of death in critically ill patients. I/R injury encompass impairment of endothelial dependent relaxation, increase in macromolecular permeability and leukocyte-endothelium interactions. Polyunsaturated fatty acids (n-3 PUFA), such as eicosapentaenoic acid (EPA, 20:5n-3) and docosahexaenoic acid (DHA, 22:6n-3) found in fish oil have several anti-inflammatory properties and their potential benefits against I/R injury were investigated using the hamster cheek pouch preparation before and after ischemia. Before the experiments, hamsters were treated orally with saline, olive oil, fish oil and triacylglycerol (TAG) and ethyl ester (EE) forms of EPA and DHA at different daily doses for 14 days. Fish oil restored the arteriolar diameter to pre ischemic values during reperfusion. At onset and during reperfusion, Fish oil and DHA TAG significantly reduced the number of rolling leukocytes compared to saline and olive oil treatments. Fish oil, EPA TAG and DHA TAG significantly prevented the rise on leukocyte adhesion compared to saline. Fish oil (44.83 ± 3.02 leaks/cm^2^), EPA TAG (31.67 ± 2.65 leaks/cm^2^), DHA TAG (41.14 ± 3.63 leaks/cm^2^), and EPA EE (30.63 ± 2.25 leaks/cm^2^), but not DHA EE (73.17 ± 2.82 leaks/cm^2^) prevented the increase in macromolecular permeability compared to saline and olive oil (134.80 ± 1.49 and 121.00 ± 4.93 leaks/cm^2^, respectively). On the basis of our findings, we may conclude that consumption of n-3 polyunsaturated fatty acids, especially in the triacylglycerol form, could be a promising therapy to prevent microvascular damage induced by ischemia/reperfusion and its consequent clinical sequelae.

## Introduction

Ischemia/reperfusion (I/R) injury is a common consequence of several clinical conditions including myocardial infarction, stroke, organ transplantation and multiple organ failure, the most prevalent cause of death in critically ill patients [1]. I/R injury is characterized by decreased endothelium-dependent relaxation in arterioles, excessive filtration of fluids in capillaries, reduction in the number of perfused capillaries, enhanced macromolecular permeability, intense inflammatory response in post capillary venules [2] and excessive reactive oxygen species (ROS) production in all segments of the microvascular tree. ROS generated by endothelial cells during reperfusion seems to function like signaling molecules, inducing P-selectin translocation to endothelial surface, leukotriene B_4_ (LTB_4_) and platelet activating factor (PAF) synthesis and activation of nuclear transcription factors, such as nuclear factor-κB (NF-κB) and activator protein-1 (AP-1). LTB_4_ and PAF cause an increased expression and activation of β_2_ integrins (CD11/CD18) on rolling leukocyte surface [3, 4], resulting in their firm adhesion to endothelium.

Another consequence of excessive ROS generation in post ischemic vessels is the inactivation of nitric oxide (NO) by superoxide ions, reducing NO bioavailability [5]. As consequence, leukocyte plugging in capillaries, leukocyte-endothelial cell adhesive interactions [6], platelet-leukocyte aggregation and albumin extravasation [7] occur. Adherent leukocytes transmigrate into interstitial compartment where they release cytotoxic metabolites and proteolytic enzymes, which induce parenchymal cell death [8] and enhance the deleterious effects of I/R.

The cardiovascular benefits of polyunsaturated fatty acids (n-3 PUFA) such as eicosapentaenoic acid (EPA, 20:5n-3) and docosahexaenoic acid (DHA, 22:6n-3) are well documented in the literature. The American Heart Association recommends the consumption of dietary supplements containing EPA and DHA ethyl esters (EE) for patients with coronary heart disease and hypertriglyceridemia [9, 10]. Moreover, they have potent immunomodulatory properties, limiting inflammation through several mechanisms, including: reduction of pro-inflammatory eicosanoids synthesis, increase in anti-inflammatory eicosanoids production, increase in resolvin synthesis (anti-inflammatory mediators involved in inflammation resolution), decrease in leukocyte chemotaxis, inhibition of adhesion molecules expression by leukocytes and endothelial cells and reduction of NF-κB activation [11].

Clinical trials have shown that the window of opportunity to reverse deleterious effects of I/R injury is very limited since the therapeutic intervention should occur within few seconds after the onset of reperfusion [1]. For this reason, there is still need of a treatment that could prevent the microvascular damage elicited by I/R [1, 12] and the consumption or ingestion of n-3 PUFA supplements could be indicated due to their anti-inflammatory properties.

In order to investigate the role of an oral treatment with n-3 PUFA against I/R damage, we have evaluated changes on arteriolar and venular diameters, number of rolling and adherent leukocytes and microvascular permeability in post capillary venules of the hamster cheek pouch preparation before ischemia and during reperfusion. We have also compared these changes to those induced by saline and olive oil treatments. Olive oil is the primary source of fat in the Mediterranean diet and its monounsaturated fatty acid (MUFA) oleic acid has also anti-inflammatory actions [13, 14]. Finally, we have compared the anti-inflammatory efficacy between TAG and EE forms of EPA and DHA in terms of reducing microvascular permeability during I/R in the same experimental conditions.

## Materials and Methods

### Ethics Statement

The experimental protocol and animal procedures were approved by the Ethical Committee of the State University of Rio de Janeiro, Brazil (H 36/94), in accordance to Guide for the Care and Use of Laboratory Animals [15].

### Animal Preparation

Experiments were performed on 191 male Syrian hamsters (*Mesocricetus auratus*, Botucatu, São Paulo, SP, Brazil) weighing 85–138 g (7–10 weeks old). All animals were fed with chow for small rodents commercially available (Nuvilab, Nuvital, Curitiba, Paraná, Brazil) and received tap water ad libitum.

Animals were distributed into 19 groups and treated orally twice a day for 14 consecutive days, at 8:00 a.m. and 5:00 p.m.

Animals treated with physiological saline (*n* = 11), olive oil (*n* = 19) and fish oil (Incromega^®^ TG3322, EPA 36 % and DHA 24 %, Croda, East Yorkshire, UK, *n* = 47) received 0.5 mL/100 g of body weight per day. For evaluation of microvascular diameter, one group was treated with fish oil at 0.2 mL/100 g/day (*n* = 6). For microvascular permeability, another group was treated with olive oil at 0.1 mL/100 g/day (*n* = 6).

**Fig. 1:**
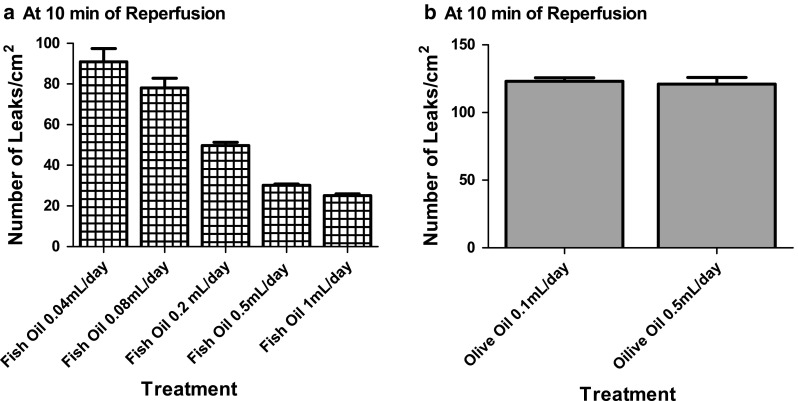
Dose response curve of fish oil and olive oil. Microvascular permeability to macromolecules was evaluated at 10 min of reperfusion, after 30 min of ischemia, in post-capillary venules of the hamster cheek pouch preparation. Animals (*n* = 6 per group) were treated orally **a** with different doses of fish oil (0.04, 0.08, 0.2, 0.5 and 1 mL per day); **b** with two doses of olive oil (0.1 and 0.5 mL per day) during 14 consecutive days

The dose of fish oil chosen for all experiments was based in a dose-response curve (fish oil × microvascular permeability). Briefly, hamsters were distributed into five groups and treated with different daily doses of fish oil (0.04, 0.08, 0.2, 0.5 and 1.0 mL) and the number of leaks/cm^2^ for each dose was recorded. The dose-response curve reached a plateau at 0.5 and 1.0 mL daily doses (Fig. 1a). As the same effect was observed with both doses, we chose the smallest one (0.5 mL/day).

We treated olive oil groups with the same daily dose of fish oil in order to warrant that both groups received the same lipid amount. Although the daily dose of olive oil (0.5 mL/day) is considered high, we did not find statistical differences in macromolecular permeability between this dose and a dose five-fold lower (0.1 mL/day) (Fig. 1b).

Sixty-six animals were equally distributed into six groups and three of these groups received EPA TAG (Omégavie^®^ EPA 90 TAG, Polaris, Pleuven, France) and the remaining three groups received DHA TAG (Omégavie^®^ DHA 90 TAG, Polaris, Pleuven, France) with the following doses: 0.004, 0.02 and 0.1 mL per 100 g of body weight per day.

Thirty-six animals were separated into six groups. Three of these groups were treated by EPA EE (Incromega^®^ EPA, Croda, East Yorkshire, UK) and the other three were treated by DHA EE (Incromega^®^ DHA, Croda, South Yorkshire, UK) at different doses: 0.004, 0.02 and 0.1 mL per 100 g of body weight per day.

### The I/R Model in the Cheek Pouch Preparation

On the experiment day (one day after the end of treatment), anesthesia was induced by an intraperitoneal injection (0.1–0.2 mL) of sodium pentobarbital (Pentobarbital sodique, Sanofi, Paris, France, 60 mg/mL) and maintained by 100 mg/kg of α-chloralose (Merck, Darmstadt, Germany) administered through a femoral vein catheter also used for fluorescein isothiocyanate (FITC)-dextran (TdB Consultancy, Uppsala, Sweden) or rhodamine-6G (Sigma Chemical Company, St. Louis, MO, USA) administrations.

Throughout surgery and subsequent experiment, the temperature of the animals was kept at 36.5 °C with a heating pad controlled by a rectal thermistor (LTB 750 Thermostat System, Uppsala process data AB, Sweden). A tracheostomy was performed to facilitate spontaneous breathing.

The cheek pouch preparation was dissected as previously described [16, 17] and mounted in an experimental chamber where it was continuously superfused, at a rate of 4.6 mL/min by a HEPES supported HCO_3_^−^-buffered saline solution (composition in mM: 110.0 NaCl, 4.7 KCl, 2.0 CaCl_2_, 1.2 MgSO_4_, 18.0 NaHCO_3_, and 15.39 HEPES Na^+^-salt) bubbled with 5 % CO_2_ -95 % N_2_ to keep the superfusate at pH 7.40 and pO_2_ at 12–15 mmHg. The temperature of the superfusion solution was maintained at 36.0 ± 0.5 °C with a circulating bath (Polyscience, model 8005, Polyscience, Niles, Illinois, USA). After 30 min of stabilization (resting period), if the preparation presented a brisk blood flow in all parts of the vascular bed including larger veins (where erythrocytes should not be discernible in the image of the blood stream), no spontaneous plasma leakage and few rolling and sticking leukocytes, a local ischemia of 30 min was performed. Ischemia in cheek pouch preparations was induced by means of a cuff, made of thin latex tubing, mounted around the proximal part of the everted pouch [18]. The cuff was placed without any visible interference of local blood flow. The intracuff pressure could be quickly increased by air compression using a syringe and rapidly decreased when required. An intracuff pressure of 200–220 mmHg resulted in a complete arrest of microvascular blood flow within a few seconds.

Before and after ischemia (reperfusion), changes in arteriolar and venular diameters, the number of rolling and adherent leukocytes and the number of leaky sites in post-capillary venules were assessed and at the end of all experiments hamsters were euthanized, under anesthesia, by an intravenous injection of potassium chloride (KCl 3 M).

### Observation of Arteriolar and Venular Diameters by Intravital Microscopy

For each cheek pouch preparation, three observation fields containing one arteriole and one venule were chosen for measurements of arteriolar and venular diameters (total magnification of 400×). The selected fields were evaluated by taking 1-min videotape recordings before ischemia, at onset, 15, 30 and 45 min of reperfusion. From videotape recordings, registration of the internal diameter of each arteriole and venule was obtained by means of an image-shearing monitor (Vista Electronics, San Diego, CA, USA, model 908). Changes in arteriolar and venular diameters were expressed as percentages, considering measurements taken before ischemia as 100 %.

### Observation of Rolling and Adherent Leukocytes by Intravital Microscopy

Circulating leukocytes were labeled by rhodamine 6G administered by an intravenous injection of 0.4 mL (0.1 mg/mL) immediately prior to observations and followed by a continuous infusion (10 μL/min) of the fluorescent dye thereafter (Syringe Pump, model 55-2222, Harvard Apparatus, Hollister, MA, USA). Fluorescent leukocytes were observed with a UV-light microscope (Leica DM LS, Leica, Wetzlar, Germany) with a set of filters (Excitation BP 546-12/Emission LP 590, Leica, Wetzlar, Germany) coupled to a closed circuit TV system (445× magnification). In each preparation, two venules (with diameters and lengths ranging from 10 to 15 μm and from 100 to 400 μm, respectively) were selected taking into account the possibility to return exactly to the same site (proximity of fat cells and bifurcations) for consecutive measurements. Experiments were performed by taking a 1-min videotape recordings of selected microvessel fields in initial control conditions (before ischemia) and subsequently at onset, 15, 30, 45 min of reperfusion.

Rolling and sticking leukocytes in post-capillary venules were counted using videotape recordings and frame-by-frame analysis. A leukocyte was considered as rolling when it was in contact with the venular wall and had a lower velocity than circulating erythrocytes and as adherent when it was immobilized at one position for at least 30 s [19].

### Macromolecular Permeability Assessment

Microvascular permeability for large molecules was quantified as the number of leaky sites (=leaks) in the area of observation. Briefly, fluorescein isothiocyanate-dextran (FITC-Dextran, MW 150,000, 50 mg/mL) was given intravenously to hamsters at 25 mg/100 mg body weight, just after the resting period. Leaks of labeled dextran were defined as visible extravascular spots (diameter > 40 µm) in post-capillary venules (internal diameter ranging from 9 to 16 μm) seen under fluorescent light using an UV-light intravital microscope (optical magnification 40×).

The number of leaks in post-capillary venules was manually scanned and counted before ischemia and during reperfusion. Maximal response to I/R occurs at 10 min after the onset of reperfusion and for this reason, this is the value reported for each experiment.

### Statistical Analysis

Results were expressed as means ± SEM. Statistical analysis was performed by one-way analysis of variance (ANOVA) followed by Tukey’s post hoc test and repeated measures by two-way ANOVA followed by Bonferroni’s post hoc test when appropriate (Graph Pad Prism 5.0 software, Graph Pad Software Inc., San Diego, CA, USA). A *P* value of less than 0.05 was considered significant.

## Results

### Changes of Microvascular Diameters Induced by Olive Oil and Fish Oil During I/R

**Fig. 2:**
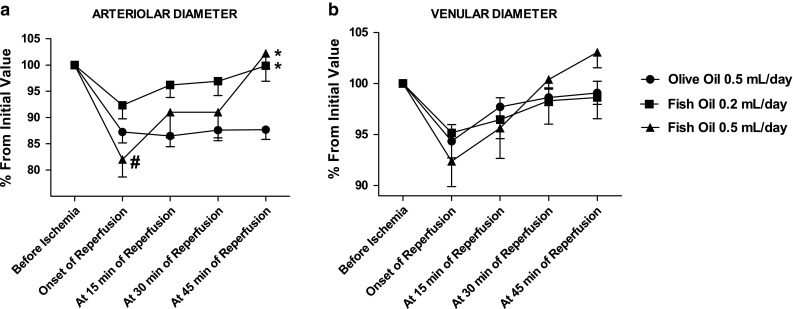
Changes in microvascular diameter induced by EPA TAG and DHA TAG treatments on the hamster cheek pouch preparation before 30 min of ischemia, at the onset, 15, 30 and 45 min of reperfusion. Animals (*n* = 6 per group) were treated orally with olive oil 0.5 mL/day and different doses of the fish oil (0.2 and 0.5 mL per day) for 14 consecutive days. **a** Changes in arteriolar diameter and **b** Changes in venular diameter. Changes in microvascular diameter are expressed as percentages, considering the measurements taken before ischemia as 100 % and are expressed as means ± SEM.^ *^Significantly different from the olive oil group at *P* < 0.05.^ #^Significantly different from fish oil (0.2 mL dose/day) treatment at *P* < 0.05

At the onset of reperfusion, olive oil and fish oil treated groups (0.2 and 0.5 mL daily doses) presented a decrease in arteriolar diameter in relation to its initial value (before ischemia). During reperfusion, the arteriolar diameter of olive oil treated hamsters remained unchanged. However, fish oil treated groups exhibited a continuous increase in arteriolar diameter, returning to pre-ischemic values at 45 min of reperfusion. At the onset of reperfusion, animals treated with fish oil 0.5 mL/day showed a significant smaller diameter compared to animals treated with 0.2 mL/day. At 45 min of reperfusion, fish oil treated groups presented a significant increase in arteriolar diameter compared to the olive oil treated group (Fig. 2a).

At the onset of reperfusion, olive oil and fish oil treated groups (0.2 and 0.5 mL daily doses) presented smaller venular diameters compared to their initial values (before ischemia). During reperfusion, olive oil and fish oil treated groups gradually increased venular diameter, returning to values near to pre-ischemic ones at 45 min (Fig. 2b).

### Protective Changes Induced by Fish Oil, EPA TAG and DHA TAG on Leukocyte Rolling During I/R

**Fig. 3:**
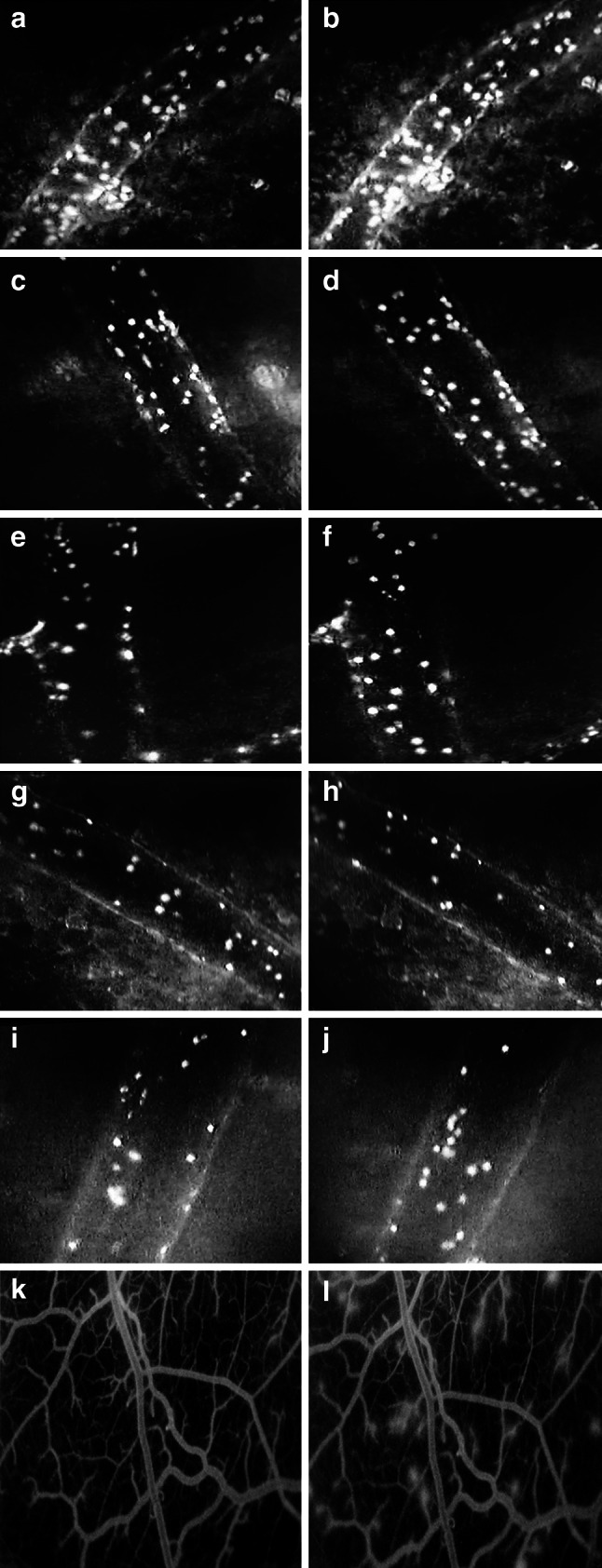
Representative photomicrographs showing leukocyte-endothelium interactions and microvascular permeability before ischemia and at 15 min of reperfusion in post-capillary venules of the hamster cheek pouch preparation. Animals were treated orally during 14 consecutive days with: **a** and **b** physiological saline 0.5 mL/day; **c** and **d** olive oil 0.5 mL/day; **e** and **f** fish oil 0.5 mL/day; **g** and **h** EPA TAG 0.1 mL/day; **i** and **j** DHA TAG 0.1 mL/day (leukocyte adhesion and rolling) and **k** and **l** physiological saline 0.5 mL/day (microvascular permeability)

Changes induced by saline, olive oil, fish oil, EPA TAG and DHA TAG on the number of rolling leukocytes during I/R are schematically represented in Fig. 3.

**Fig. 4:**
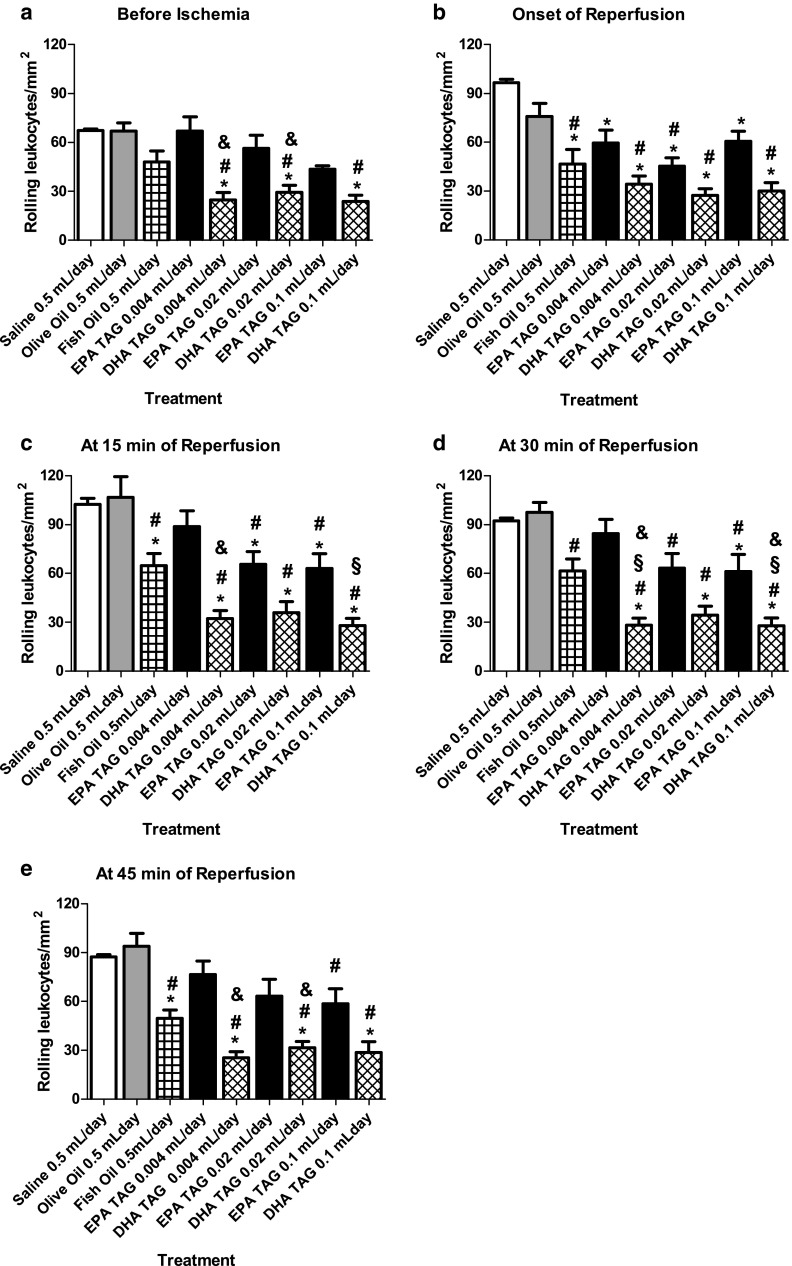
Changes in the number of rolling leukocytes/mm^2^/min, in post-capillary venules, of the hamster cheek pouch preparation, before 30 min of ischemia, at the onset, and 15, 30 and 45 min of reperfusion. Animals (*n* = 5 per group) were treated orally with physiological saline 0.5 mL/day, olive oil 0.5 mL/day, fish oil 0.5 mL/day and different doses of the triacylglycerol form of EPA and DHA (0.004, 0.02 and 0.1 mL per day) during 14 consecutive days. **a** Number of rolling leukocytes/mm^2^/min before 30 min of ischemia, **b** at the onset of reperfusion, **c** at 15 min of reperfusion, **d** at 30 min and **e** at 45 min of reperfusion. Data are expressed as means ± SEM.^ *^Significantly different from physiological saline at *P* < 0.05.^ #^Significantly different from olive oil group at *P* < 0.05.^ §^Significantly different from the fish oil group. ^&^Significantly different from the EPA group at the same daily dose at *P* < 0.05

Under basal conditions (before ischemia), DHA TAG treatments at all doses significantly reduced the number of rolling leukocytes compared to physiological saline and olive oil. We could also show that DHA TAG significantly decreased leukocyte rolling when compared to corresponding doses of EPA TAG. There were no significant differences between fish oil and saline, olive oil, EPA TAG and DHA TAG treatments (Fig. 4a).

At the onset of reperfusion, the treatment with fish oil, EPA TAG and DHA TAG at all doses significantly prevented the rise in the number of rolling leukocytes in comparison to physiological saline treatment. On the other hand, olive oil failed to prevent the elevation in the number of rolling leukocytes. Treatments with fish oil, EPA TAG at 0.02 mL and DHA TAG at all doses significantly reduced leukocyte rolling compared to olive oil (Fig. 4b).

At 15 min of reperfusion, treatments with fish oil, EPA TAG at 0.02 and 0.1 mL and DHA TAG at all doses significantly inhibited the rise in the number of rolling leukocytes in comparison to physiological saline. Fish oil, EPA TAG at 0.02 and 0.1 mL and DHA TAG at all doses significantly reduced the number of rolling leukocytes compared to olive oil treatment. However, olive oil and EPA TAG (0.004 mL) treatments were not able to inhibit the elevation in the number of rolling leukocytes. DHA TAG at 0.1 mL significantly decreased the number of rolling leukocytes when compared to fish oil. It was also shown that at the same daily dose (0.004 mL), DHA TAG significantly reduced the number of rolling leukocytes compared to EPA TAG (Fig. 4c).

At 30 min of reperfusion, treatment with EPA TAG 0.1 mL DHA TAG at all doses significantly prevented the rise in the number of rolling leukocytes compared to physiological saline. Daily treatments of olive oil, fish oil, EPA TAG at 0.004 and 0.02 mL failed to prevent the elevation in the number of rolling leukocytes compared to physiological saline. EPA TAG at 0.02 and 0.1 mL and DHA TAG at all doses significantly reduced the number of rolling leukocytes compared to olive oil treatment. DHA TAG treatments (at 0.004 and 0.1 mL) significantly reduced the number of rolling leukocytes when compared to fish oil treatment. We have also observed that with the same daily doses (0.004 and 0.1 mL), DHA TAG decreased significantly the number of rolling leukocytes compared to EPA TAG (Fig. 4d).

At 45 min of reperfusion, treatment with fish oil and DHA TAG at all doses significantly prevented the elevation in the number of rolling leukocytes in comparison to physiological saline. However, treatments of olive oil and EPA TAG (at all doses) were not able to inhibit the rise in the number of rolling leukocytes compared to physiological saline. Fish oil, EPA TAG at 0.1 mL and DHA TAG at all doses significantly decreased the number of rolling leukocytes compared to olive oil. At same daily doses (0.004 and 0.02 mL), DHA TAG elicited a significant reduction in the number of rolling leukocytes compared to EPA TAG (Fig. 4e).

### Protective Changes of Fish Oil, EPA TAG and DHA TAG on Leukocyte Adhesion During I/R

Changes induced by saline, olive oil, fish oil, EPA TAG and DHA TAG on the number of sticking leukocytes during I/R are schematically represented in Fig. 3.

**Fig. 5:**
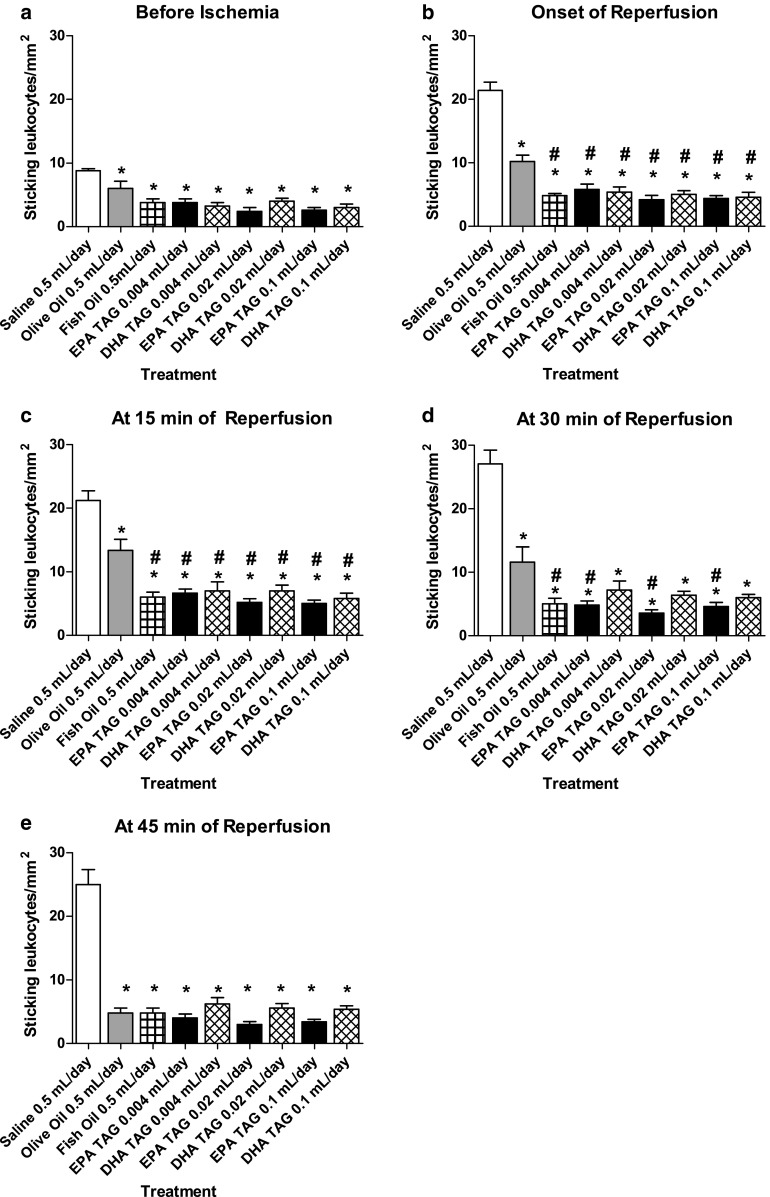
Changes in the number of sticking leukocytes/mm^2^/min, in post-capillary venules of the hamster cheek pouch preparation before 30 min of ischemia, at the onset of reperfusion and at 15, 30 and 45 min after reperfusion. Animals (*n* = 5 per group) were treated orally with physiological saline 0.5 mL/day, olive oil 0.5 mL/day, fish oil 0.5 mL/day and different doses of the triacylglycerol form of EPA and DHA (0.004, 0.02 and 0.1 mL per day) during 14 consecutive days. **a** Number of adherent leukocytes/mm^2^/min before 30 min ischemia, **b** at onset of reperfusion, **c** at 15 min of reperfusion, **d** at 30 min and **e** at 45 min of reperfusion. Data are expressed as means ± SEM.^ *^Significantly different from physiological saline at *P* < 0.05.^ #^Significantly different from olive oil at *P* < 0.05

In control conditions (before ischemia), the number of adherent leukocytes is significantly lower in olive oil, fish oil, EPA TAG and DHA TAG treated groups compared to physiological saline-treated group (Fig. 5a).

At the onset of reperfusion, olive oil, fish oil, EPA TAG and DHA TAG treatments significantly prevented the rise in the number of sticking leukocytes in comparison to physiological saline treated animals. We have also demonstrated that fish oil, EPA TAG and DHA TAG treatments significantly reduced the number of adherent leukocytes compared to olive oil (Fig. 5b).

At 15 min of reperfusion, olive oil, fish oil, EPA TAG and DHA TAG treated groups exhibited significant reduction in the number of adherent leukocytes in comparison to physiological saline treated group. Fish oil, EPA TAG and DHA TAG treatments significantly reduced the number of sticking leukocytes compared to olive oil treatment (Fig. 5c).

At 30 min of reperfusion, olive oil, fish oil, EPA TAG and DHA TAG treated groups presented significantly lower number of sticking leukocytes compared to physiological saline. Daily treatments of fish oil, EPA TAG at all doses significantly reduced leukocyte adhesion compared to olive oil (Fig. 5d).

At 45 min of reperfusion, olive oil, EPA TAG and DHA TAG treated groups exhibited significant reduction in the number of sticking leukocytes in relation to physiological saline. The number of adherent leukocytes on fish oil, EPA TAG and DHA TAG treated groups was not statistically different from olive oil treated group (Fig. 5e).

### Protective Changes Induced by Fish Oil, EPA TAG, EPA EE and DHA TAG on Microvascular Permeability During I/R

**Fig. 6:**
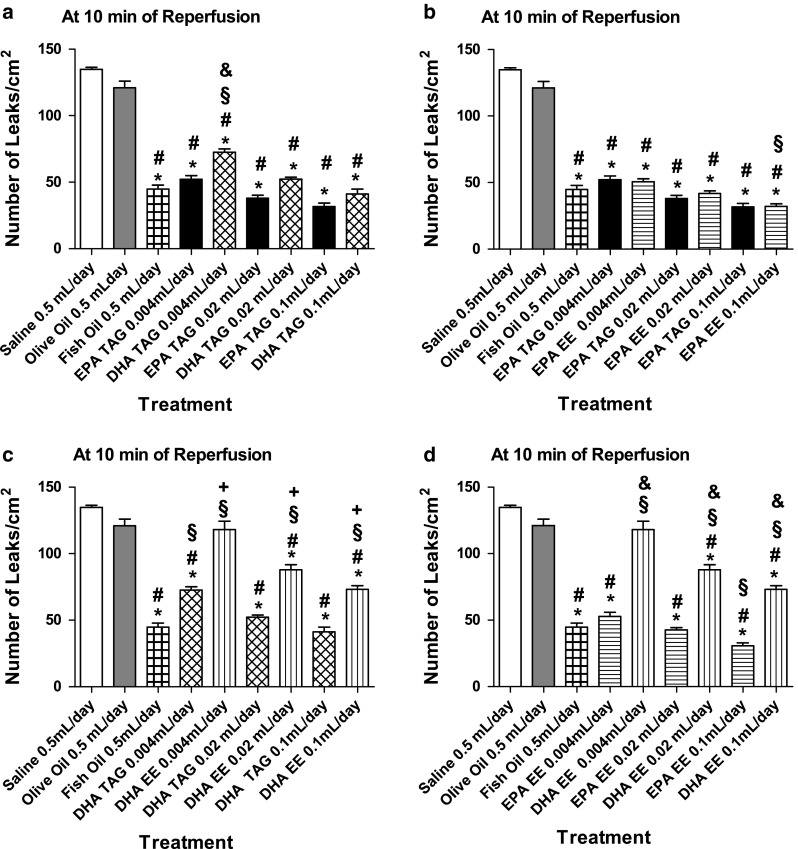
Changes in microvascular permeability, in post-capillary venules, of the hamster cheek pouch preparation at 10 min of reperfusion. Animals (*n* = 6 per group; except for the olive oil group, *n* = 8) were treated orally with physiological saline 0.5 mL/day, olive oil 0.5 mL/day, fish oil 0.5 mL/day and different doses of triacylglycerol (TAG) and ethyl ester (EE) forms of EPA and DHA (0.004, 0.02 and 0.1 mL per day) during 14 consecutive days. **a** Maximal number of leaks/cm^2^ comparisons between physiological saline, olive oil, fish oil, EPA TAG and DHA TAG groups, **b** Maximal number of leaks/cm^2^ comparisons between physiological saline, olive oil, fish oil, EPA TAG and EPA EE groups, **c** Maximal number of leaks/cm^2^ comparisons between physiological saline, olive oil, fish oil, DHA TAG and DHA EE groups, **d** Maximal number of leaks/cm^2^ comparisons between physiological saline, olive oil, fish oil, EPA EE and DHA EE groups. Data are expressed as means ± SEM.^ *^Significantly different from physiological saline at *P* < 0.05.^ #^Significantly different from olive oil at *P* < 0.05.^ §^Significantly different from fish oil at *P* < 0.05.^ &^Significantly different from EPA at the same daily dose at *P* < 0.05.^ +^Significantly different from DHA TAG at same daily dose at *P* < 0.05

Daily treatment with fish oil and all doses of the TAG form of EPA and DHA significantly prevented the increase in the number of leaks/cm^2^ induced by I/R compared to physiological saline and olive oil treatments. Moreover, fish oil significantly prevented the increase in leaky sites/cm^2^ in comparison to DHA TAG at 0.004 mL/day. At the same daily dose (0.004 mL), EPA TAG elicited a significant reduction in the number of leaky sites/cm^2^ compared to DHA TAG (Fig. 6a).

Treatment with fish oil and EPA at all doses in both forms (TAG and EE) significantly prevented the elevation in the number of leaks/cm^2^ compared to physiological saline. Treatment with EPA at all doses in both forms significantly inhibited the rise in the number of leaks/cm^2^ compared to olive oil treatments. EPA EE 0.1 mL/day significantly reduced the number of leaky sites/cm^2^ compared to fish oil treatment (Fig. 6b).

Treatment with fish oil, DHA TAG at all doses and DHA EE at 0.02 mL and 0.1 mL/day doses significantly prevented the rise in the number of leaks/cm^2^ in comparison to physiological saline and olive oil. Treatment with DHA EE at 0.004 mL/day failed to inhibit the increase in macromolecular leakage. DHA TAG (at 0.004 mL/day) and DHA EE (at all doses) treated animals presented significantly greater number of leaks/cm^2^ in comparison to fish oil treated animals. At same daily doses, DHA TAG treatment significantly reduced the macromolecular leakage compared to DHA EE treatment (Fig. 6c).

Daily treatment with fish oil, EPA EE and DHA EE at all doses (except 0.004 mL/day) significantly prevented the rise in the number of leaks/cm^2^ compared to physiological saline and olive oil. Fish oil treatment significantly inhibited the increase in leaks/cm^2^ in comparison to DHA EE treatment at all doses. However, fish oil treated group exhibited a significantly higher number of leaky sites/cm^2^ in relation to the EPA EE 0.1 mL/day treated group. The treatment with EPA EE decreased the number of leaky sites/cm^2^ more effectively than DHA EE. At same daily doses, EPA EE treatments elicited a significant reduction in the number of leaks/cm^2^ compared to DHA EE treatment (Fig. 6d).

## Discussion

The novel findings of the present study were: (a) Fish oil (but not olive oil) restored arteriolar diameter to control (pre ischemic) values during reperfusion; (b) Fish oil, EPA TAG and DHA TAG limited leukocyte-endothelium adhesive interactions and macromolecular permeability increase elicited by I/R; (c) DHA TAG prevented the elevation of the number of rolling leukocytes more effectively than fish oil and EPA TAG and (d) EPA EE inhibited the increase in microvascular permeability induced by I/R more effectively than DHA EE.

I/R injury is known to compromise endothelium dependent relaxation and to elicit an intense inflammatory response in post-capillary venules. The changes in microvascular diameter in consequence of reduced endothelial dependent vasodilation can be assessed on arterioles and venules. Conversely, leukocyte rolling and adhesion and macromolecular permeability can be assessed preferably in post-capillary venules. Under acute stimulus (such as 30-min ischemia) leukocytes tend to roll and adhere on venular and not on arteriolar endothelium. Similarly, disruption of the endothelial barrier, manifested by increase in microvascular permeability, is a phenomenon that also occurs preferably in post capillary venules. For this reason, microcirculatory inflammatory variables, shown in this study, such as leukocyte rolling and adhesion as well as macromolecular permeability were evaluated only in post-capillary venules.

**Fig. 7:**
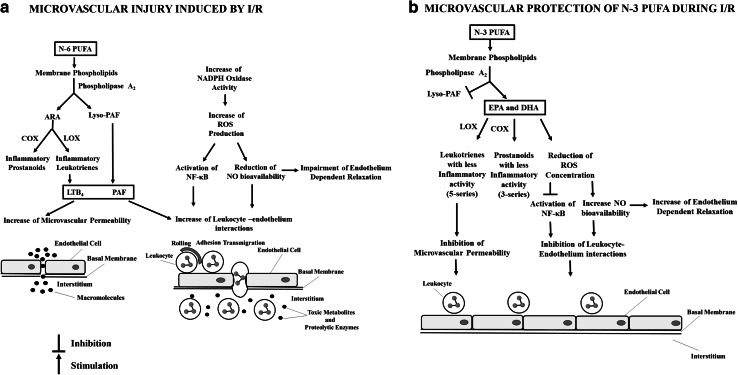
Proposed mechanisms for ischemia/reperfusion injury and for microvascular protection elicited by n-3 polyunsaturated fatty acids (n-3 PUFA). **a** During ischemia/reperfusion, phospholipase A_2_ is activated and releases lyso PAF factor), the precursor of platelet activating factor (PAF) and arachidonic acid (ARA) from membrane phospholipids of leukocytes and endothelial cells. ARA is then converted to pro-inflammatory prostaglandins by cyclooxygenase (COX) and leukotrienes by (lipoxygenase). Leukotriene B_4_ (LTB_4_) and PAF elicit the disruption of the endothelial cell barrier with a consequent increase in macromolecular permeability. PAF and LTB_4_ also induce an increase in leukocyte-endothelium interactions. Simultaneously, an increase in nicotinamide adenine dinucleotide phosphate (NADPH) oxidase activity occurs in endothelial cells and in leukocytes. The excessive ROS production causes a depletion of NO and activates the nuclear factor-κB (NF-κB). Reduced NO bioavailability results in an impairment of endothelium dependent vasodilation, an increase in leukocyte rolling, adhesion and transmigration. In the interstitium, the leukocytes release toxic metabolites and proteolytic enzymes, which induce parenchymal cell death; **b** Chronic oral treatment with n-3 PUFA results in their incorporation in membrane phospholipids. The lyso-PAF generation is inhibited, interrupting the pathway for PAF synthesis. PLA_2_ releases only eicosapentaenoic acid (EPA) and docosahexaenoic acid (DHA). Subsequently they are converted to prostanoids and leukotrienes with less inflammatory activities by COX and LOX, respectively. In the absence of PAF and LTB_4_, reduction of microvascular permeability occurs. EPA and DHA downregulate NADPH oxidase activity and neutralize ROS directly, reducing ROS concentration. A decrease in ROS concentration inhibits NF-κB activation and increases NO bioavailability. Consequently, an improvement of endothelium dependent vasodilation, inhibition of leukocyte-endothelium interactions and subsequent tissue damage occur

It is well documented in the literature that EPA and DHA are able to modulate immune responses [20]. Increased consumption of n-3 PUFA result in their incorporation to membrane phospholipids inhibiting the metabolism of n-6 PUFA arachidonic acid (ARA, 20:4n-6), reducing the synthesis of ARA-derived pro-inflammatory eicosanoids [21] in platelets, endothelial cells and leukocytes [22, 23]. In addition, EPA can act as a substrate for cyclooxygenase (COX) and lipoxygenase (LOX), giving rise to eicosanoids with less biological activities: 3-series prostaglandins and thromboxanes and 5-series leukotrienes [20, 24]. On the other hand, EPA and DHA oxidation originates a family of lipid mediators, with potent anti-inflammatory properties, named resolvins (resolution phase interaction products) and protectins [20, 25, 26]. Resolvins and protectins affect the signal transduction induced by cytokines and interfere with ROS production (mainly hydrogen peroxide) that activate directly NF-κB enhancing the expression of several pro-inflammatory molecules, including Interleukin (IL)-1, IL-6, IL-8, tumor necrosis factor-α (TNF-α), E-selectin and vascular cell adhesion molecule-1 (VCAM-1) [27]. In addition, EPA and DHA can activate NF-κB directly [28]. The beneficial changes of n-3 PUFA during I/R injury are summarized on Fig. 7.

A common feature of I/R injury is the decrease in endothelium-dependent vasodilation, as a consequence of NO depletion caused by ROS. NO is also an endogenous regulator of leukocyte rolling and adhesion in post-capillary venules [5]. During I/R, reduced bioavailability of NO upregulates the expression of adhesion molecules by endothelium. n-3 PUFA, due to their double bond rich structure, have high susceptibility to oxidation and can directly neutralize ROS in situ [24]. Moreover, n-3 PUFA can also decrease superoxide anions production in neutrophils via prostaglandin dependent pathway [29] and decrease nicotinamide adenine dinucleotide phosphate (NADPH) oxidase activity [30, 31]. Based on these studies, we have hypothesized that the increase in venular diameter during reperfusion (Fig. 2b) could affect the number of adherent leukocytes in olive oil and fish oil groups, but these numbers remained practically unaffected during reperfusion (Fig. 5). The observed increase in venular diameter caused reduction of leukocyte rolling only at 45 min of reperfusion in hamsters treated with fish oil. Regarding olive oil treatment, the number of rolling leukocytes remained high during reperfusion (Fig. 4).

At the onset of reperfusion, the release of flow elevates intraluminal pressure and causes stretching of the vascular wall, which, in turn, reacts with contraction evidencing a typical intrinsic property of the vessels, the so called myogenic response [32]. The myogenic response, in vivo, is much more prominent in arterioles [33] than in venules [34, 35]. More recently, studies revealed [36, 37] that the myogenic response is dependent on hydrogen peroxide (via thromboxane A_2_ receptor) in pressurized arterioles and small veins from rat *gracilis* skeletal muscle. It was also shown that myogenic tone was significantly attenuated by catalase that inactivates hydrogen peroxide [37]. In our study, we expected that fish oil treatment could diminish hydrogen peroxide concentration (which is high during I/R) and thus the myogenic response in arterioles and venules, but it did not occur (Fig. 2a, b). The plausible explanation for this difference is the experimental preparation: the cheek pouch is an in vivo preparation and the vascular environment is not so well controlled as in isolated vessels preparations suggesting that other factors and the resulting complex interaction among them must be involved in the regulation of the myogenic response. During reperfusion, fish oil (but not olive oil) treatment restored the arteriolar diameter to pre ischemic values, possibly by two mechanisms: increase in NO bioavailability and activation of large conductance Ca^2+^-dependent K^+^ (BK_Ca_) channels. Studies have shown that fish oil enhances the activity of nitric oxide synthase [38, 39], prevents NO quenching and directly activates BK_Ca_ channels [40].

I/R injury is characterized by leukocyte-endothelial cell interactions [41], which involves three distinct steps: rolling, firm adhesion of leukocytes and transmigration [42].

Leukocyte rolling depends on the expression of a class of adhesion molecules named selectins that include L-selectin, constitutively expressed on leukocyte surface, E-selectin, found on cytokine activated endothelial cell surface, and P-selectin, expressed on activated endothelial cells and platelets [43]. In the present investigation, we showed that chronic oral treatment with MUFA oleic acid present in olive oil has no effect on leukocyte rolling. However, chronic oral treatment with fish oil, EPA TAG and DHA TAG significantly inhibited the increase in leukocyte rolling compared to chronic oral treatment with saline. In addition, our data demonstrated that chronic oral treatment with DHA TAG was the most important modulator of this phenomenon among n-3 PUFA tested (Fig. 4). In fact, some in vitro studies have demonstrated that DHA is more effective than EPA in terms of inhibit leukocyte rolling. Pre-incubation with DHA reduced significantly monocyte rolling [44, 45] and P and E-selectin expression on activated endothelial cell cultures [46, 47]. Another study has shown that incubation with EPA was unable to inhibit the expression of E-selectin on TNF-α stimulated endothelial cells culture [48]. It seems that in our preparation, leukocyte rolling induced by reperfusion injury was more dependent on P-selectin than on L- or E-selectin expression and that DHA inhibited its expression on endothelium and platelets. Firstly, because P-selectin is rapidly translocated to the endothelial surface after injury and according to Kanwar and co-workers [49] it is critical for leukocyte rolling induced by I/R.

Second, E-selectin is synthetized de novo and its expression on the endothelial surface begins within 90–120 min after the stimulus [50], and our experiments were not long enough to observe its participation in leukocyte rolling. Third, EPA derived Resolvin E1, augmented L-selectin shedding from leukocytes [25] and if leukocyte rolling in our experimental conditions was mediated by L-selectin, EPA should be more effective in preventing the rise in the number of rolling leukocytes than DHA, but it was not observed. Finally, it was shown elsewhere that DHA incubation inhibited the expression of P-selectin induced by TNF-α on endothelial cell culture [46].

Firm adhesion of leukocytes to the endothelium is mediated by integrins expressed on the leukocyte surface and members of immunoglobulin superfamily, such as intercellular adhesion molecule-1 (ICAM-1) and VCAM-1, presented on endothelial cell membrane [51]. I/R injury induces the release of LTB_4_ and PAF which in turn upregulate the expression and activate β_2_ integrins (CD11/CD18) on the rolling leukocyte surface [3, 4] promoting their firm adhesion to the endothelium. In our study, chronic oral treatments with fish oil, EPA TAG and DHA TAG, exhibited equivalent potencies, significantly prevented the elevation of the number of sticking leukocytes compared to physiological saline and olive oil chronic oral treatments (except at 45 min of reperfusion) (Fig. 5). Probably it was due to their capacity to downregulate directly or indirectly the expression of integrins and adhesion molecules from the immunoglobulin superfamily. EPA-derived LTB_5_ is less potent in upregulating CD11b/CD18 expression [52] than LTB_4_. Moreover, EPA-derived Resolvin E1 downregulates the expression of CD18 [25] on the leukocyte surface. EPA and DHA inhibit the generation of endothelial cell PAF on the activated endothelium, thus decreasing monocyte adhesion [53]. In addition, incubation with DHA can reduce the expression of ICAM-1 and VCAM-1 on endothelial cells and diminish the adhesion of monocytes and neutrophils to the endothelium monolayer in vitro [47, 53, 54]. Incubation with EPA decreased the expression of ICAM-1 and VCAM-1 on activated endothelial cells from EA.hy926 lineage [48] and inhibited monocyte adhesion to activated endothelial cells culture [55].

During I/R, leukocytes release factors that may disrupt the endothelial barrier. Activation of leukocytes via β_2_ integrins stimulates the release of soluble factors, such as LTB_4_, which produces endothelial cytoskeletal rearrangement, gap formation and increase in microvascular permeability [8, 56], In the present study, fish oil, EPA TAG, EPA EE and DHA TAG inhibited significantly the rise in the number of leaky sites compared to chronic oral treatments with physiological saline and olive oil (Fig. 6a, b).

Furthermore, it was shown that chronic oral treatment with both forms of EPA inhibited the increase in macromolecular permeability more effectively than chronic oral treatment with both forms of DHA (Fig. 6a, d). EPA TAG and EPA EE decreased macromolecular leakage with similar effectiveness (Fig. 6b). However, the TAG form of DHA was more potent in terms of inhibiting microvascular permeability than its EE form (Fig. 6c). Since PAF and LTB_4_ have been reported to increase vascular permeability [57–59], and n-3 PUFA incorporation to membrane phospholipids have been shown to suppress PAF synthesis in endothelial cells [44] and monocytes [60] and give origin to a less potent leukotriene, the LTB_5_ it is conceivable that EPA TAG, DHA TAG and EPA EE are effectively incorporated into leukocyte and endothelial cell membrane phospholipids, culminating with decreased formation of leaky sites in response to I/R. In contrast, DHA EE was not able to prevent the increase in microvascular permeability. The less anti-inflammatory potency of this form of DHA is probably due to its ineffective incorporation to membrane cell phospholipids.

We tested crescent doses of EPA and DHA, in both forms and did not observe a dose response curve, as expected. The responses presented an all-or-none pattern, mainly regarding leukocyte rolling and adhesion. A plausible explanation for this phenomenon, is that leukocyte rolling and adhesion are processes dependent on distinct classes of molecules. For example, DHA TAG is effective at preventing leukocyte rolling, probably by inhibition of P-selectin translocation to endothelial cells membranes or by induction of L-selectin shedding from the leukocyte surface while EPA did not affect leukocyte rolling possibly because it did not interfere with selectins shedding and translocation. Concerning inhibition of leukocyte adhesion, our data indicated that DHA and EPA are equipotent, probably because both decrease the expression of ICAM-1 and VCAM-1 by the endothelium. Regarding the slight difference between the doses, it seems that the lowest of EPA and DHA doses were already enough to diminish the number of rolling and sticking leukocyte and any additional amount ingested was not able to enhance their beneficial changes on these variables.

This is the first study that clearly stated the forms of EPA and DHA used, evaluated the efficacy of the TAG form in terms of mitigating microvascular injuries elicited by ischemia reperfusion (via reduction of leukocyte-endothelial cell adhesive interactions and macromolecular permeability) and compared the effectiveness of their TAG and EE forms through assessment of microvascular permeability.

On the basis of our findings, we may conclude that continuous consumption of n-3 polyunsaturated fatty acids, especially in the triacylglycerol form, may be a promising therapy to prevent microvascular damage induced by ischemia/reperfusion and its consequent clinical sequelae.

## List of Abbreviations

ANOVA: Analysis of variance
AP-1: Activator protein-1
ARA: Arachidonic acid (20:4n-6)
BK_Ca_: Large conductance Ca^2+^-dependent K^+^ channels
COX: Cyclooxygenase
DHA: Docosahexaenoic acid (22:6n-3)
EE: Ethyl ester
EPA: Eicosapentaenoic acid (20:5n-3)
FITC: Fluorescein isothiocyanate
ICAM-1: Intercellular adhesion molecule-1
IL: Interleukin
I/R: Ischemia/reperfusion
LOX: Lipoxygenase
LT: Leukotriene(s)
MUFA: Monounsaturated fatty acid(s)
NF-κB: Nuclear factor-κB
NADPH: Nicotinamide adenine dinucleotide phosphate
PAF: Platelet activating factor
PUFA: Polyunsaturated fatty acid(s)
Resolvins: Resolution phase interaction products
ROS: Reactive oxygen species
TAG: Triacylglycerol
TNF-α: Tumor necrosis factor-α
VCAM-1: Vascular cell adhesion molecule-1
